# Indents

**Published:** 1905-02-15

**Authors:** 


### INDENTS.

☞ **Brief Articles of Technical or Scientific Value will receive notice and comment. Contributions invited.**

**Toothache.**

R. Menthol,
Chloral ............aa dram ss.
Gum Camphor ......grs. xv.
Cocaine ...............grs. iij.
Mix and triturate to liquification.
Sig. Apply to tooth cavity on cotton.

**Malaria and Grip Cold.**

R. Sulphate of Quinine,
Acetanelid .........aa dram j.
Caffein ..............grs. xij.
Mix and make into twelve capsules.
Sig. Take a capsule three times each day, one before each morning and noon meal and the other at bedtime.

**Anti-Neuralgic Remedy.**

R. Acetanelid ........ounces j ss.
Bicarbonate of Soda..drams vj.
Carbonate of Ammonia,
Citrate of Caffein...aa drams iij.
Mix and pulverize finely and bottle securely.
Sig. Give five grain doses, and not more than three doses in two hours.

**Mouth Wash.**

R. Alcohol .................oz. j.
Eucalyptus Oil
Menthol.
Thymol .............aa dramj.
Mix and dissolve in ten ounces of alcohol to which has boen added twenty ounces of distilled water containing three percent boric acid. Color to suit.—Dr. Rousell, in February *Cosmos*.

**A Peculiar Accident.** A patient of a Springfield dentist swallowed a bur while his teeth were being filled. Later, by the aid of an X-ray machine, the bur was located in the œsophagus, from which it was removed by forceps.

**Vulcanizer Explodes.** The explosion of a vulcanizer in the dental office of Drs. G. E. Mann and S. E. Gates, Cincinnati, caused severe injuries to both dentists. They were at work with their heads bent over the vulcanizer when the explosion occurred, and their faces were badly burned.

**Nitrous Oxid and Gasoline.** Dr. Gautz, in the February *Cosmos*, relates a personal encounter that came very near to a serious accident. He was using liquid nitrous oxide to get a blowpipe blast, for fusing platinum solder with a gasoline flame, when the apparatus exploded. The Doctor says he will not mix these agents again, and advises caution to any one who thinks of doing so.

**For Cleaning The Teeth.** The addition of a small portion of Euthymol Tooth Paste to pumice stone for cleansing teeth at the dental chair offers a valuable adjunct to treatment.

It imparts an agreeable flavor, is cooling and stimulating to the mucus membrane, prevents the pumice stone from being thrown about, and is gratefully received by the patient.—Goodman A. Miller, D.D.S., Chicago, Ills.

**Fermentable Foods.** In general, sticky, doughy, semi-soluable substances will be found most injurious to the teeth as they adhere to the teeth and lodge in the crevices where they ferment and supply caries-producing agents. Cooking food renders it more fermentable and oticky, and contribute much to the universal existence of caries among civilized people.—W. D. Miller, in Dental *Cosmos*.

**Acidity from Food.** In mixture of saliva with food, especially bread, a more active fermentation takes place and greater acidity is produced than in simple solutions of carbohydrates in bouillon or saliva. The intensity and rapidity of fermentation in ordinary food debris lodged about the teeth must therefore be very great, and is practically independent of the small amount of soluable carbohydrates which may exist in the saliva.—W. D. Miller, in *Cosmos.*

**Caries, Reasons for Susceptibility.** First, structural deficiency in calcification which Dr. Black has shown fallacious. Second, environment is thought to be a potent reason; the physical and chemical character of the saliva, and its possible bactericidal action; the nature of the food; the shape of the teeth and their positions in the arch, etc. Third, diathesis; certain physical or pathogenetic conditions predisposing to caries. In my judgment this third reason is tenable only so far as it influences the other two.—W. D. Miller, in *Cosmos.*

**Epithelioma from Dental Operation.** Dr. Ballock, in the *Cosmos*, cites a case of epithelioma, the history of which traces back to a dental operation. A fissure of the lip, caused by the carelessness in extracting, did not heal and underwent epitheliomatous degeneration. The Doctor suggests that care should be taken to avoid accidents of this kind when working for patients with such a diathesis and especially when forty years or over old. Epithelioma originates from some irritant, and a wound of this character in a patient so disposed would be liable to develop in epithelioma.—February *Cosmos.*

**Wire Rubber Dam Ligatures.** Dr. Wm. Mitchell in the December *Items of Interest* suggests using a fine, soft wire, doubled and tightly twisted as a means of forcing the dam and gum out of cavities extending deeply under the gum, such as the ordinary clamps will not successfully accomplish. Wax or varnish the wire to make the dam stick to it. After adjusting the dam pass the wire between and around the tooth and twist the ends until it is closely adopted to the neck of the tooth, cut off the surplus ends and force dam and wire above cavity margin with a thin chisel.

**Acid or Alkaline Saliva.** When food stuffs, which are impregnated with alkaline saliva, lodge between the teeth or in cavities of decay, a more rapid development of bacteria will take place than when the reaction of the saliva is acid, the result being that the alkali is soon neutralized and a degree of acidity brought about equal to or slightly in excess of that produced where the saliva had an initial acid reaction. Therefore the chances of caries taking place in a mouth which is generally alkaline in saliva are about as favorable as in one where the saliva is acid.—W. D. Miller, in *Cosmos*.

**Judgment and Skill in the Construction of Bridge-work.** Good judgment is best acquired by first obtaining a knowledge of correct principles, and by observation in the application of these principles to practical cases; noting reasons for all failures in securing satisfactory results. The acquirement of skill is a gradual development by practice. Personal adaptability, and ability to comprehend conditions and to successfully execute details are essential factors in the acquirement of skill. Judgment and skill in design and execution will develop accordingly as these fundamental principles are cultivated. A safe rule for the novice is to be conservative rather than radical.—H. J. Goslee, in *Items*.

**Greased Teeth Immune to Caries.** J. Sim Wallace says: I have made numerous observations which seem to me to indicate that the relative freedom from caries among people who eat much fat is out of all proportion to what one might expect considering fat as neutral in its action on the teeth. Nor do I think that we can account for this freedom from caries in fat-eaters by the fact that such people may eat relatively less of the carbohydrates. Miller thinks the facts act as protectors against the acids. He instances the fact that isolated teeth having no antagonists are little used and become coated with a greasy coating which seems to protect them from decay.

**Caries of the Teeth.** Generally speaking we recognize two stages in the destruction of the tooth by caries: first, the decalcification of the hard part of the tooth; second, the dissolution of the decalcified organic matrix. Decalcification is done by acids produced chiefly by the fermentation of the carbohydrates in the mouth; the dissolution of the organic matrix is a result of the digestive action of ferments of the nature of trypsin produced by bacteria. In enamel there is but one process and the second stage is lacking because of the small amount of organic matrix.—W. D. Miller, in *Cosmos*.

**Tooth Bleaching.** The quickest way to bleach a tooth that I know of, when it has not been discolored by alloys, copper, amalgam, essential oils or gum resins, is by using aluminum chlorid and Labarraque's solution. The next is by using powdered alum and moistening with Labarraque's solution. And the next is by moistening the interior of the tooth with dilute sulfuric acid and then filing in sodium dioxid; and last, but not least, is the 25 percent pyrozone method. Pure terebene (C 10 H 16) will slowly dissolve oils. It should be sealed in the tooth and the application removed once a week.—Dr. Miller, in *Cosmos*.

**Replacing a Bridge Tooth.** Dr. Wm. Hirschfeld reported a method of replacing a broken tooth upon a permanent bridge in the mouth. The bridge in question was perfectly made and could not be removed without ruining it. It was necessary to replace a tooth immediately. The thickness of the bar made it difficult to drill the usual holes for the new tooth pins; besides it would have weakened the bar. He finally decided to solder a flat tooth to a strip of gold plate of the width of the tooth and space, and long enough at each end to permit of their being bent around, one above and the other below the bar, thus clamping the latter—the bar of the bridge, of course, being first properly fitted for this clamp, and the band or clamp backing of the tooth being perfectly adjusted to the bar and strengthened by solder at the angle nearest the articulating portion of the tooth before final fixing.—*Cosmos.*

**Returns from Philippines.** Maj. Rob. T. Oliver, formerly an Indianapolis dentist, but now supervising dental surgeon in the regular army, is visiting in that city after an absence of nearly four years in the Philippine Islands.

Maj. Oliver is accompanied by his wife and little son, Robert Oliver, Jr., born in the Islands two and a half years ago. Maj. Oliver is on his way to West Point, where he has been assigned on transfer from the Philippine service.

While in the islands Maj. Oliver had charge of twenty of the thirty men in the dental corps. He says the work of the corps has been of great value to the service, and that since its work began, 76,000 individual cases have been handled, 83,000 operations having been performed.

Maj. Oliver's talk of his travels and sight-seeing, and of the work of the Philippine sanitary commission, is interesting, and gives a good idea of what Americans are accomplishing in the far East.—*American Journal.*

**Splint for Pyorrhoea Teeth.** Dr. Gordon White, in the December *Items*, describes a unique method of making a metal splint for holding loose teeth firmly together. Take a plaster impression of the lingual and also one of the buccal surfaces, including a portion of the occlusal surfaces in each case, of the bicuspids and molar teeth for instance of either side or jaw, as the case may be. From the impressions make dies and swedge metal plates of gold or other material, to fit and include the teeth desired. Attach these two plates—one covering the lingual surfaces and the other covering the buccal surfaces—by small iridio-platinum wires passing from one plate to the other over the grinding surfaces of the teeth, locating each wire so that it will pass in the proximal space so as to permit occlusion of the teeth. A splint made in this manner can be sprung to place and will hold the teeth firmly, thus preventing the irritation to the inflamed gums and periosteum. The splint can also be readily removed for cleansing the teeth.

**Transmission Of Palatal Defects.** Mr. R. C. Lucas says: "In the Transactions of the Clinical Society for 1888 will be found a paper in which I showed that a congenital absence of the lateral teeth incisor was an indication that a hare-lip or cleft palate in the same place might follow in a successive generation. I now for the first time make public a further observation in relation to this deformity. It is that a feebly-developed lateral incisor on one side in the mother may foreshadow a hare-lip and cleft palate in her offspring. I have twice noted this association and I exhibit to the society a photograph and drawing of the teeth of a mother, showing this defective lateral incisor, whose first and only child has hare-lip. Hare-lip and cleft-palate are frequently associated with other deformities which apparently present no causative relation. I mentioned that in the family with supernumerary fingers and toes which I recorded, hare-lip and cleft-palate had also appeared in the later generations. Roux has recorded a similar association."—*Record.*

**To Sharpen Gates-Glidden Drills.** With the use of a piece of oilstone shaped like a pocket-knife blade (say three inches in length and with a sharp edge), it is a comparatively easy matter to bring in turn each blade of the drill to an edge—examining the drill frequently during the process under a magnifying glass.

It is essential to good work that the stone edge be kept sharp. To accomplish this, obtain a piece of hard wood, say three inches long and two inches in width, making sure that it is straight and perfectly level. Now take a strip of sheet lead two inches wider than the wood, but the same length, or longer, so that it may be continued over each end; tack it over the sides and ends (if necessary) to the wood, having adjusted it evenly. Upon the surface thus formed sprinkle some No. 1 emery, and rub the stone lengthwise without oiling or wetting the surface. To keep the stone in perfect condition this process should be continued after every dozen drills. As to the time required for sharpening the stone, three minutes usually suffices.—Dr. Fernandez, in *Dental Cosmos*.

**Dentalone.** Messrs. Parke, Davis & Co. have added another valuable preparation to their long list of useful and efficacious medicinal compounds. The newcomer is "Dentalone," which is supplied in one-ounce special glass-stoppered vials.

Dentalone represents a saturated solution of the well-known analgesic and local anesthetic, chloretone, in a liquid vehicle composed of oil of cloves, oil of gaultheria and oil of cassia. It is therefore a powerful obtundent and anesthetic and its principal field of usefulness will be in the treatment of exposed pulps, sensitive dentine, sensitive gums and other pathologic states of which "toothache" is a prominent symptom.

Dentalone is a strictly ethical preparation. It is made exclusively for the use of the dental profession and will not be sold in the drug stores as an ordinary tooth ache remedy. We are confident that a trial of dentalone will convince the dental practitioner of its superiority to preparations containing cocain. It is not harmful in its action upon the tissues, and its effect is thorough and lasting. It should fill a conspicuous niche in the office cabinet of every dentist in the land.

